# Reorientation of single-wall carbon nanotubes in negative anisotropy liquid crystals by an electric field

**DOI:** 10.3762/bjnano.7.74

**Published:** 2016-06-08

**Authors:** Amanda García-García, Ricardo Vergaz, José F Algorri, Gianluigi Zito, Teresa Cacace, Antigone Marino, José M Otón, Morten A Geday

**Affiliations:** 1 CEMDATIC, E.T.S.I. Telecomunicación, Universidad Politécnica de Madrid, Avda. Complutense 30, 28040 Madrid, Spain; 2 GDAF-UC3M, Departamento de Tecnología Electrónica, Universidad Carlos III de Madrid, Butarque 15, Leganés, 28911, Spain,; 3 CNR-ISASI and Physics Department, University of Naples Federico II, Via Cinthia Monte S. Angelo, 80126, Naples, Italy

**Keywords:** Anchoring, carbon nanotubes, impedance, liquid crystal, negative anisotropy, Raman spectroscopy, reorientation, single-wall CNTs

## Abstract

Single-wall carbon nanotubes (SWCNT) are anisotropic nanoparticles that can cause modifications in the electrical and electro-optical properties of liquid crystals. The control of the SWCNT concentration, distribution and reorientation in such self-organized fluids allows for the possibility of tuning the liquid crystal properties. The alignment and reorientation of CNTs are studied in a system where the liquid crystal orientation effect has been isolated. Complementary studies including Raman spectroscopy, microscopic inspection and impedance studies were carried out. The results reveal an ordered reorientation of the CNTs induced by an electric field, which does not alter the orientation of the liquid crystal molecules. Moreover, impedance spectroscopy suggests a nonnegligible anchoring force between the CNTs and the liquid crystal molecules.

## Introduction

Single-wall carbon nanotubes (SWCNTs) are highly anisotropic nanoparticles (NPs) formed from a single, wrapped graphene sheet. SWCNTs may be metallic or semiconductive, depending on the rolling angle (chirality) and diameter [1]. The SWCNT one-dimensional structure leads to an anisotropic conductivity, where the charge transport is favored along the longitudinal axis [2].

Liquid crystals (LCs) are self-organized anisotropic fluids, well-known for their use in flat panel mobile, PC, and TV displays. Besides consumer electronic products, LCs are employed in many photonic devices such as spatial light modulators, beam steerers, and tunable lenses and filters [3–4]. LCs are usually confined into thin (few µm) cells, where an adequate conditioning of the inner cell surfaces leads to long-range orientational order [5], represented by a vector called the director. In *homogenous* alignments, the director lies in the plane of the cell surfaces, while in *homeotropic* alignment the director orients perpendicular to the plane of the cell surfaces.

LCs feature, amongst others, electrical, magnetic, and optical (birefringence) anisotropy properties. In the absence of an external field, the LC orientation in thin cells is dictated by the anchoring forces of the conditioned surfaces. However, the orientation may be altered if an external (electric) field above a certain threshold voltage (*V*_th_) is applied. As a result, the effective permittivity of the LC material varies with the applied voltage. The LC director reorients to minimize the system energy; this energy depends on the anchoring forces of the substrate, the elastic parameters of the LC, and the applied field [6].

Calamitic LCs – the most commonly used LC in all kinds of applications – are rod-like uniaxial molecules having different properties along the long axis (which roughly matches the director orientation) or perpendicular to the long axis. They are customarily classified as positive and negative LCs according to their dielectric anisotropy [6]. In a positive LC material, the dielectric constant along the director is larger than the degenerate dielectric constant perpendicular to the director. Consequently, the molecules tend to align with an applied electrical field. In a negative LC material the opposite is the case.

Thus, a homogenously aligned, positive LC sandwiched between two electrodes will reorient when a sufficiently large electric field is applied, as will a homeotropically aligned, negative LC, but in the inverse direction.

SWCNTs immersed in a LC produce changes in the elastic and dielectric constants [7] and in the electro-optic properties [8–11] of the LC. This feature has attracted much attention since it opens for the possibility of tuning the LC properties by doping with SWCNTs. In the last years, several studies have described SWCNT orientation in LC matrices, based on for example the conductivity of SWCNT-doped LC cells at different switching degrees [12–15].

SWCNT–LC blends co-align spontaneously while filling or relaxing in a homogeneously aligned cell, due to the intrinsic anisotropy of both components. In positive LC cells [15–16], the SWCNTs and the LC both switch and relax together.

On the other hand, SWCNTs dispersed in liquids also align according to the electric field [17]. This rise the question of two possible contributions to the SWCNT alignment, namely the co-alignment by induced by the LC matrix and the electro-static forces exerted directly on the SWCNTs.

In a previous paper [18] we determined the electrical behavior of positive LC cells doped with CNTs, and the results were in accordance with a reorientation of the nanotubes due to the electrical field, as well as the irreversible condition of this orientation in some cases. But since a positive liquid crystal and the CNTs reorient in the same manner under an electrical field, it was not possible to separate the switching of the CNTs caused by the interaction with the field and with the LC molecules in this study.

Therefore, this study is focused on determining the origin of the SWCNT switching in a LC matrix, by isolating the LC switching and the SWCNT switching, and by studying both individual SWCNTs in a negative LC matrix and the macroscopic impedance of a liquid crystal cell filled with negative LC doped with SWCNTs. In order to separate the field induced switching of the LC and the SWCNTs, a cells with homogenously aligned LC of negative dielectric anisotropy is employed.

The SWCNT switching pattern in this matrix is studied both microscopically, using Raman spectroscopy and microscopic imaging on micrometer-sized areas of the cells, as well as macroscopically, using impedance measurements averaging the whole cell response. Micro-Raman spectroscopy is a powerful technique that allows label-free identification of molecular species and their conformation or orientation by virtue of their vibrational fingerprint. It can be used efficiently in soft matter and biological applications, providing spatially resolved information [19–20]. Here, we apply an inverted micro-Raman setup (WiTec 300), whose description can be found in [21], to track the orientation of the LC component and CNTs as a function of the external electric field.

These complementary studies result in a complete study of the system and confirm the hypothesis under consideration, that is, that SWCNTs reorient in the presence of an electric field even in an oppositely oriented LC matrix. When a positive LC is doped with SWCNTs, the SWCNTs reorient together with the LC, as previously studied by impedance measurements [15].

A negative dielectric, anisotropic, nematic, calamitic LC presents the same properties as a positive, calamitic LC (orientational order, anchoring forces, etc). It has been shown that negative nematics orient SWCNTs in the same way as positive nematics, that is, along the director [12].

However, if a negative nematic LC is homeotropically aligned, it does not reorient when an electric field is applied perpendicularly to the substrate and thus does not assist the SWCNT reorientation.

**Figure 1:**
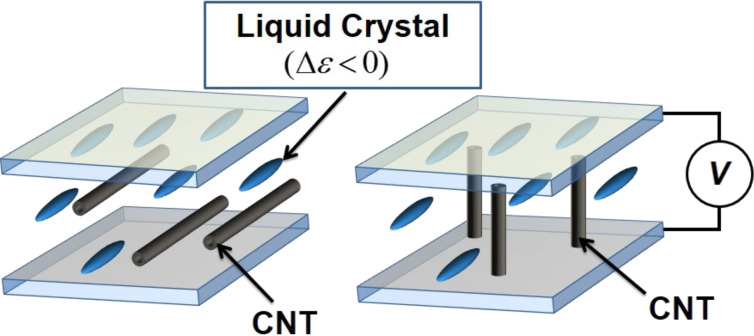
SWCNT-doped negative LC reorientation hypothesis: the LC remains homogeneously aligned while SWCNTs reorient with the applied electric field.

In this way, LC anchoring forces towards SWCNTs are present but without any reorientation effect (Figure 1).

## Raman measurements

**Figure 2:**
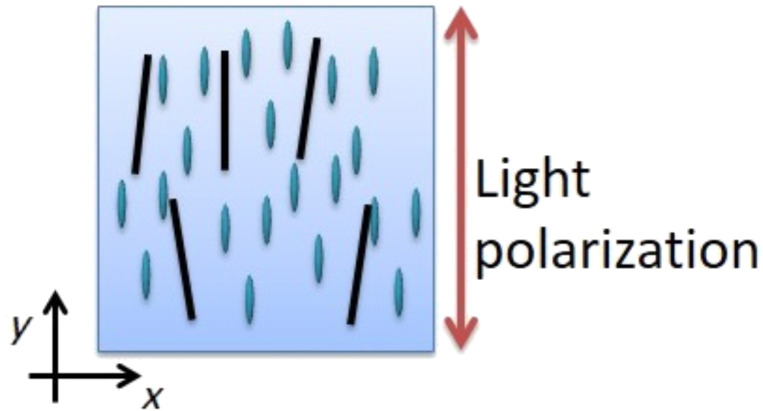
Linear polarized light direction for Raman measures at *V* = 0.

Schymura and Scalia [22] noted the change in the SWCNT Raman band intensity as a function of the applied electric field, showing that a SWCNT reorientation from planar to perpendicular. Due to the anisotropic properties of SWCNTs, the polarized Raman intensity received along the longitudinal axis is higher than that along the transversal axis. Thus, the change in the SWCNT Raman spectrum intensity indicates a variation in the SWCNT orientation (Figure 2).

In the present work, it is expected that the Raman peaks caused by the LC do not vary due to the stable position of the LC, while the Raman peaks correlated to the SWCNTs decrease with an increasing applied voltage to the cell, when they change their orientation.

The SWCNT Raman spectrum is formed by several bands with the most relevant being: the radial breathing mode (RBM), located around 250 cm^−1^, which is related to the SWCNT diameter [23]; the G-band (around 1600 cm^−1^); and the G’-band (around 2700 cm^−1^), which is an overtone of the D-band (due to structure imperfections) [24]. The profile of the G-band indicates whether the SWCNT under study is semiconductive (one single peak with a Lorentzian profile) or metallic (one split peak with a Breit–Wigner–Fano profile).

## Impedance measurements

Impedance measurements provide information about the charge mobility in a volume, that is, the conductivity properties of a system. The impedance (*Z*) is a complex number formed by two terms: magnitude (|*Z*|) and phase, which depends on the frequency. The impedance result at a given frequency shows whether the material behaves as an electrical energy storage system (i.e., a capacitor with an impedance phase value of approximately −90°) or as a conductor (phase value around 0°) with a specific resistivity value (related with the impedance magnitude).

As a LC cell is formed by two parallel electrodes with a dielectric material between them (the LC), it may be described in impedance terms as a capacitor, where capacitance depends on the dielectric constant of the LC. The presence of SWCNTs (being conductive nanoparticles) in the LC media may change the impedance of the system. Their reorientation from planar to the substrate (i.e., the longitudinal axis parallel to the electrodes) to perpendicular (i.e., the longitudinal axis is perpendicular to the electrodes) may be detected as a LC−SWCNT cell conductivity increase. This is attributed to the favored charge transport between the electrodes through the SWCNT longitudinal axis. The SWCNTs may even form conductive paths that may connect the two electrodes. This effect would lead to a dramatic impedance decrease (i.e., conductivity increase) and the change of the equivalent electrical behavior of the system would change from a capacitor to a resistor due to the possible direct electrode connection.

## Results and Discussion

### Switching mechanism

CNTs in dielectric media may be reoriented by an externally applied field [25]. The reorientation of the CNTs is caused by a combination of anisotropic polarizability (probably caused by separation of the ionic charges on the CNT surface) and on the dielectrophoretic effect, in which the permutation of the electrical field caused by the CNT itself leads to an overall alignment [26].

**Figure 3:**
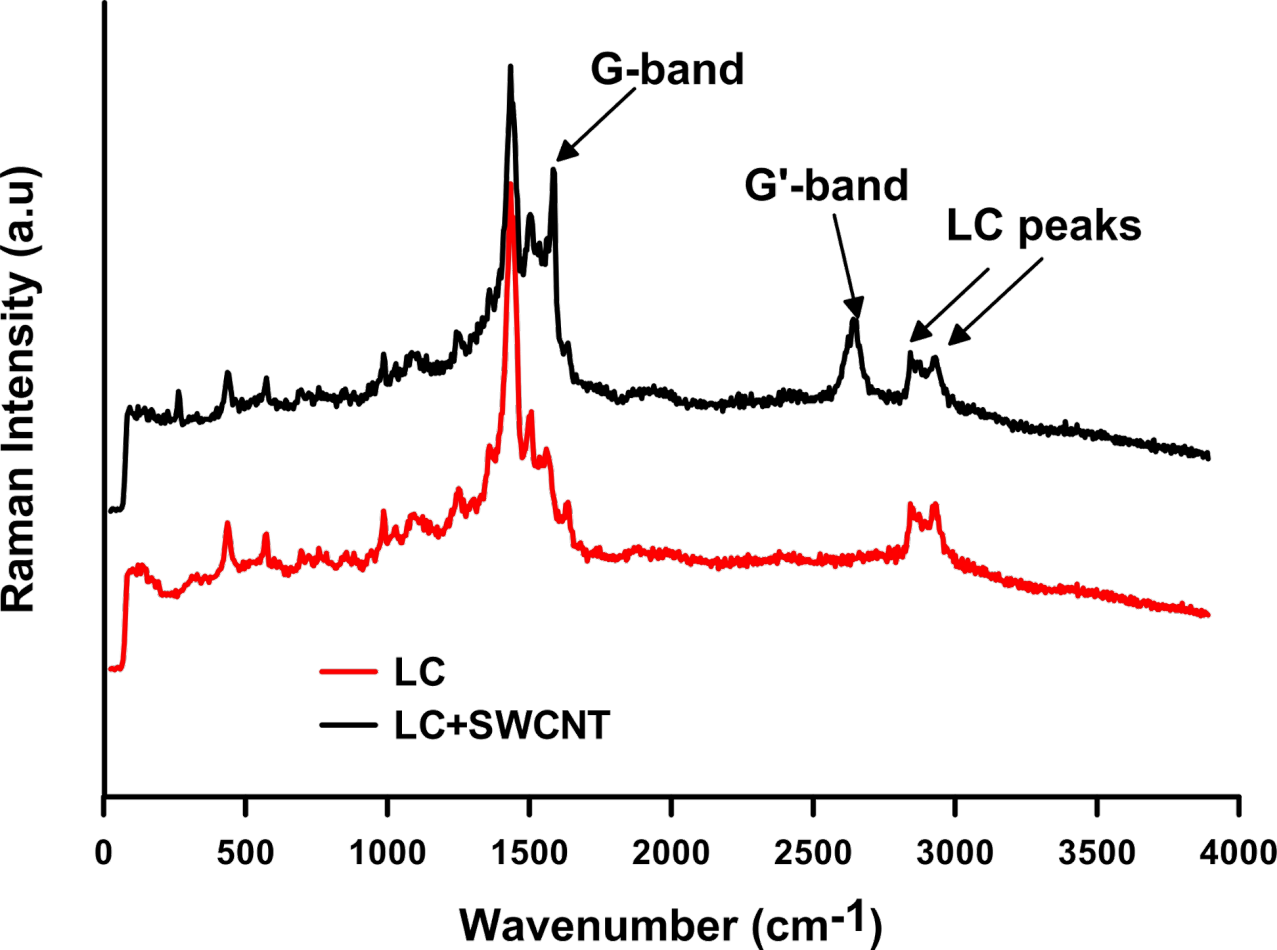
Raman spectra of a SWCNT-doped LC cell (λ = 523 nm). The G and G’-bands are related to SWCNTs. The profile of the G-band should show a small peak in the case of metallic behavior. However, the Raman signal from the LC obstructs this peak.

The small spot diameter of the visible wavelength Raman spectroscopy source (2 µm) allows for high spatial resolution measurements. Scanning the focused light beam over the surface of the SWCNT-doped LC cell allows for the detection of the individual nanotubes or nanotube aggregates. The Raman spectra of the SWCNTs and LC device are studied to find the peaks related to each. The peaks at 1592 cm^−1^ (G-band) and 2647 cm^−1^ (G’-band) belong to SWCNTs [22]. In order to detect the LC peaks, undoped cells with the same alignment configuration were studied. These studies show that the LC detected peaks are at 2843 cm^−1^ and 2932 cm^−1^ (Figure 3).

Often, SWCNTs are visible by standard microscopic imaging because they form large aggregates when the dispersion mixture is not perfect. However, there are areas where individual SWCNTs are perfectly dispersed or present in submicroscopic aggregations of a low number of nanotubes that are not visible through the microscopic image. These regions are identified by Raman spectroscopy as the SWCNT-band peaks detectable (Figure 3).

**Figure 4:**
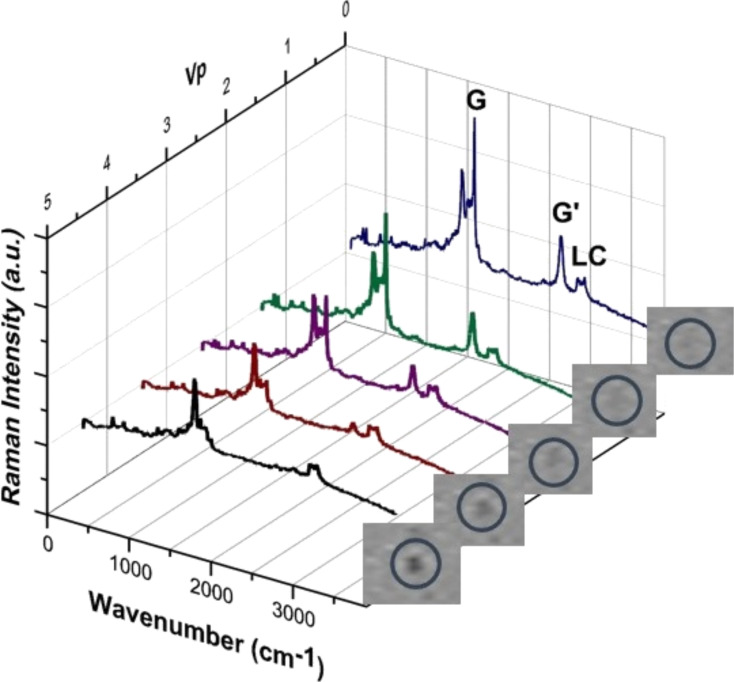
Raman spectrum at different driving voltages (0 V, 1.5 V, 2.5 V, 3.5 V and 4.5 V). The microscope images confirm the light scattering difference between un-switched (0 V) and switched state (5.5·*V*_p_).

The SWCNT Raman peaks decrease when the electric field is applied to the cell and disappear at voltages greater than 5.5·*V*_p_ (*V*_p_, peak voltage). This indicates a SWCNT reorientation from planar to perpendicular, variable with the applied electric field. Due to the alignment characteristics explained above, the LC molecules do not reorient when the electric field is applied. As can be appreciated from the Raman spectrum (Figure 4), the LC peaks do not change when a field is applied.

Thus the SWCNTs reorient due to the effect of the electric field and the anchoring force of the LC molecules is not strong enough to overcome it. The visual difference in the appearance of the point under study is observed simultaneously with the Raman measurement by microphotography (Figure 4, right). The SWCNTs parallel to the LC cell surface are invisible because the LC molecules anchored to the SWCNTs are parallel to the surrounding LC molecules, and the volume of the SWCNT itself is imperceptible. This appearance changes with the SWCNT reorientation, and an increase of the scattering is clearly visible in the photograph. The light scattering in the excited state is caused by the LC molecule disorder around the reoriented SWCNT complex.

**Figure 5:**
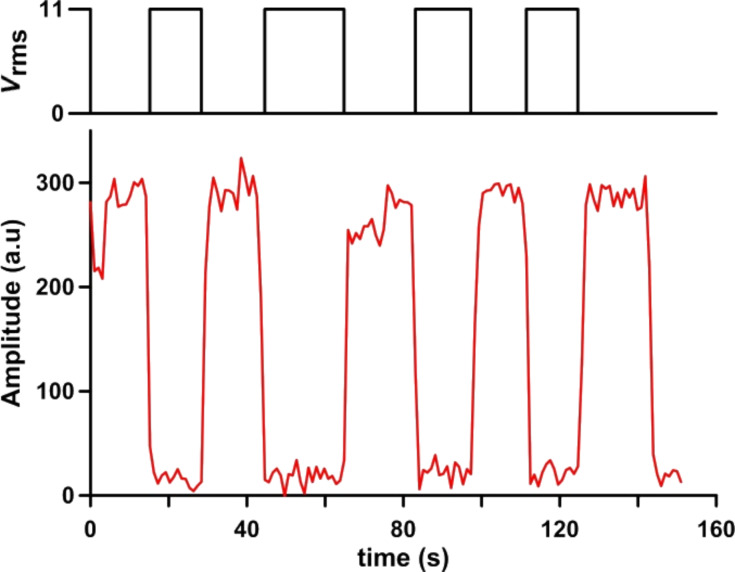
Raman G-band amplitude variation (below) during a driving voltage sequence between 0 and 11*V*_rms_ (above).

In some areas, the SWCNT switching is reversible. Several points were studied by applying an on–off square signal (0 – 11*V*_rms_; V_rms_ – root mean square voltage). The Raman G-band amplitude changes are synchronized with the signal applied (Figure 5). The G-band is visible in the unbiased state, becoming practically imperceptible at 11*V*_rms_. In every cycle of the applied signal, the Raman G-band amplitude returns to its initial value when the driving voltage is suppressed.

**Figure 6:**
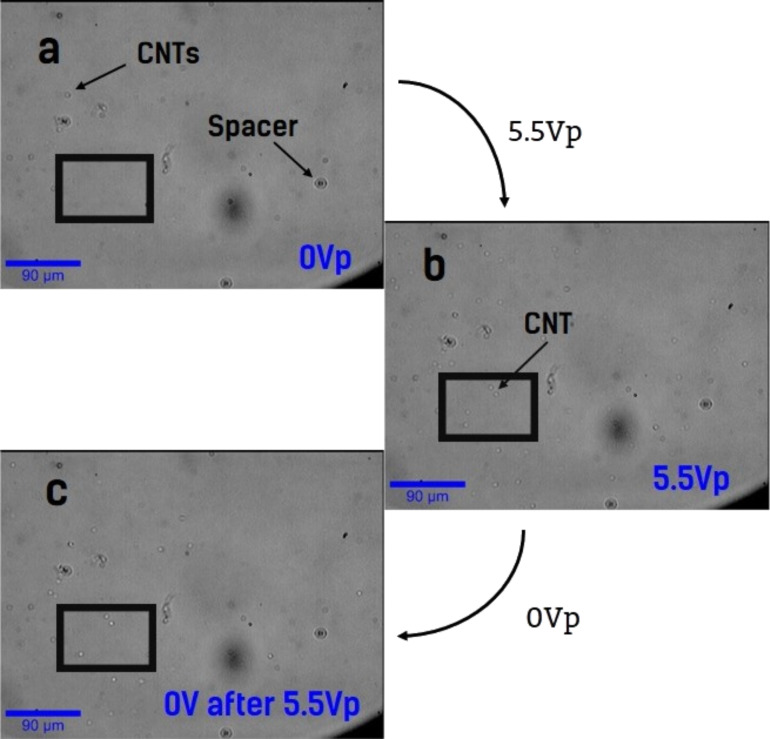
Cell images under microscopic study during a complete off–on–off voltage cycle. Sample surface (a) prior to voltage application, (b) at 5.5*V*_p_ and (c) at 0*V*_p_ after several off–on (maximum 5.5*V*_p_) cycles. The squares highlight four points: from the (b) to the (c) condition, one of them relaxes while the other three do not recover the original state.

However, in other areas, the relaxation of the SWCNT orientation is gradually lost, and yet in others, no relaxation of the SWCNT orientation is detected at all (Figure 6).

This lack of reversibility can be linked to agglomeration, or imperfect dispersion, of the nanotubes. The agglomerates may become so large that the anchoring force that the LC exerts is not sufficient to realign the agglomerate, or they may even become encrusted in the alignment layers.

**Figure 7:**
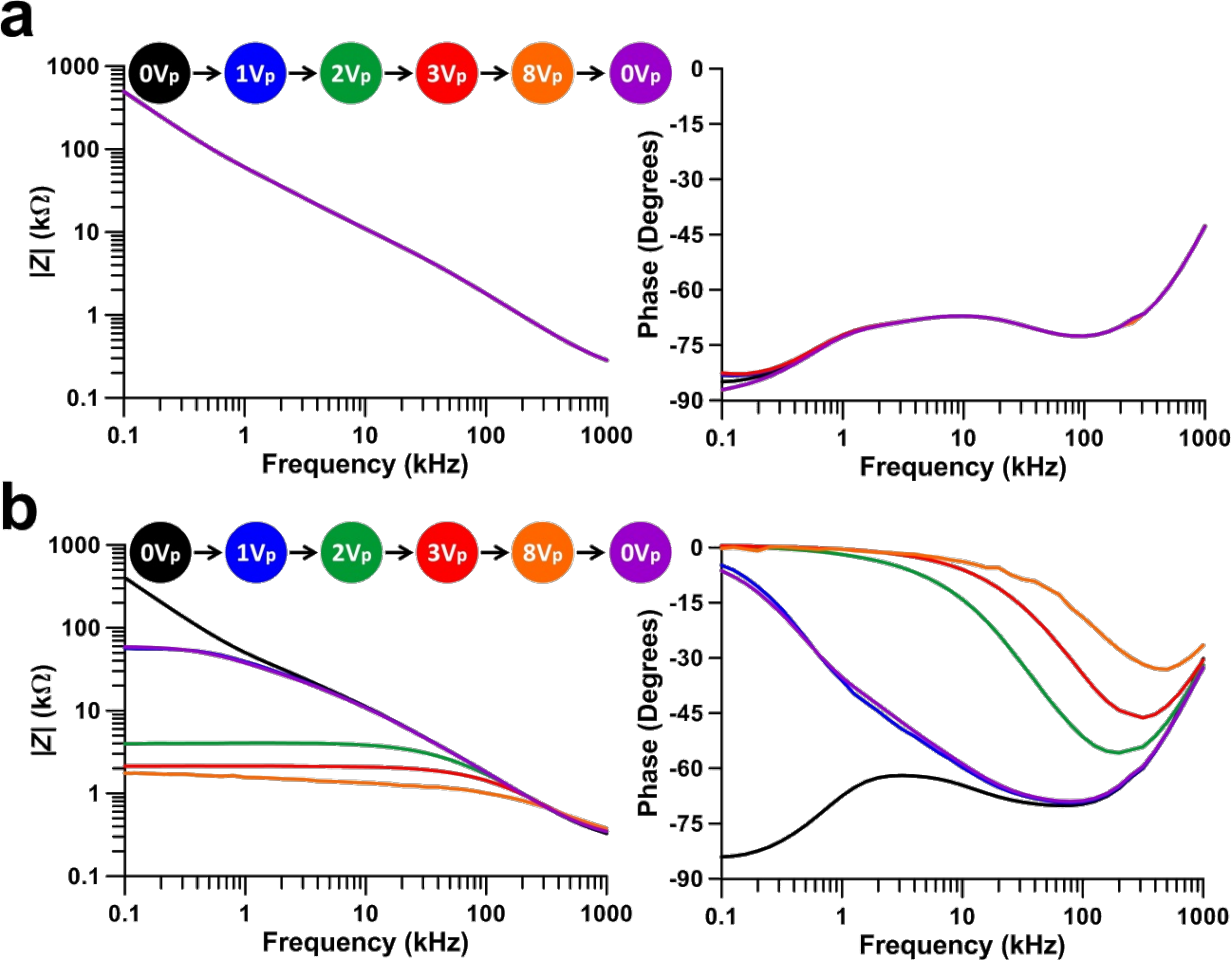
Impedance magnitude and phase measurements of (a) undoped and (b) SWCNT-doped LC cells.

Thus, as not all the SWCNTs recover to the planar alignment, the system behavior is not fully reversible from a macroscopic point-of-view. The impedance measurements are in accordance with this fact. The undoped LC cell shows invariable impedance behavior with the applied electric field (Figure 7a) because the LC molecules do not change their position, and there is not any change of their effective dielectric permittivity.

The SWCNT-doped LC cell impedance is variable according to Figure 7b. In an initial unbiased state (0 V), the electrical behavior is close to that of a capacitor, and similar to that of the undoped cell. This result seems to be caused by the position of the majority of the SWCNTs, that is, parallel to the substrate. The impedance magnitude decreases (from 0.5 MΩ to approximately 2 kΩ at 100 Hz) when the bias field is applied to the SWCNT-doped LC cell. The conduction increases as a consequence of the alignment of the longitudinal axis of the nanotubes with the electric field in concordance with the conclusions of the Raman results. The electrical behavior changes from that of a capacitor, to a resistor, especially at midrange frequencies (from 100 Hz to 5 kHz). The impedance does not recover the original value when the bias voltage is removed (return to 0 V). In fact, the measurement result is nearly the same as at 1*V*_p_. The SWCNT-doped LC cell does not present fully reversible behavior in the impedance study, in agreement with the Raman measurements. This could be caused by SWCNTs that do not recover their original planar orientation. As these SWCNTs are in a position perpendicular to the contacts, the electron transport is facilitated by the longitudinal axis.

### Threshold voltage

In order to determine a potential threshold voltage for the SWCNT switching, a detailed study of impedance variation between 0 V and 4*V*_p_ in steps of 0.1*V*_p_ was performed.

**Figure 8:**
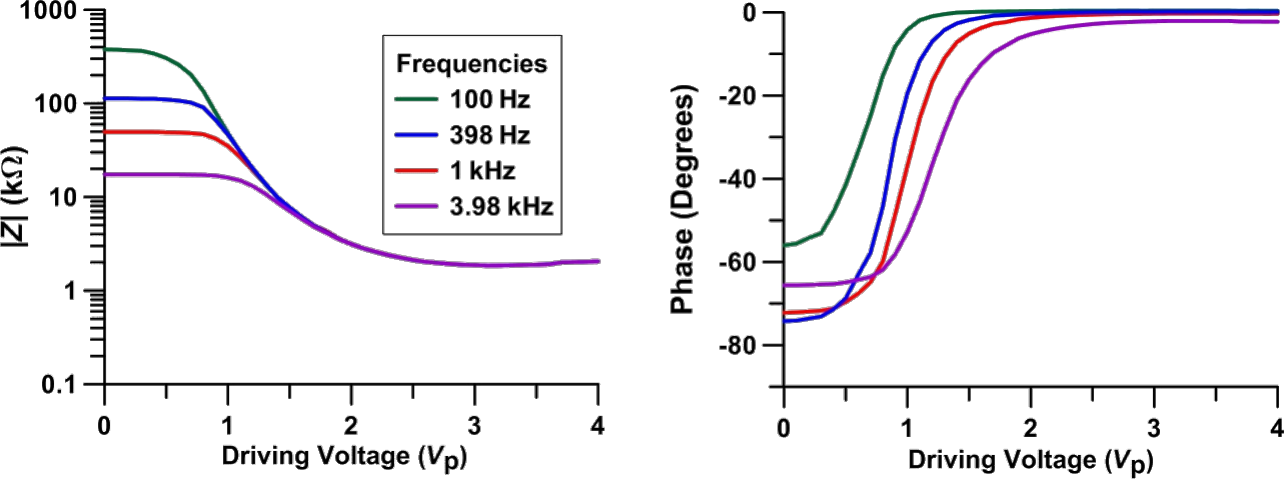
Impedance magnitude and phase variation at different frequencies as a function of applied driving voltage, *V*_p_. The LC is in a nematic phase.

The results show a constant value in the impedance up to 0.7*V*_p_ (Figure 8). Both the magnitude and phase of impedance change smoothly with voltage for values below 0.7*V*_p_. Thus, on a macroscopic level, 0.7*V*_p_ appears to be the SWCNT-doped LC cell threshold voltage.

**Figure 9:**
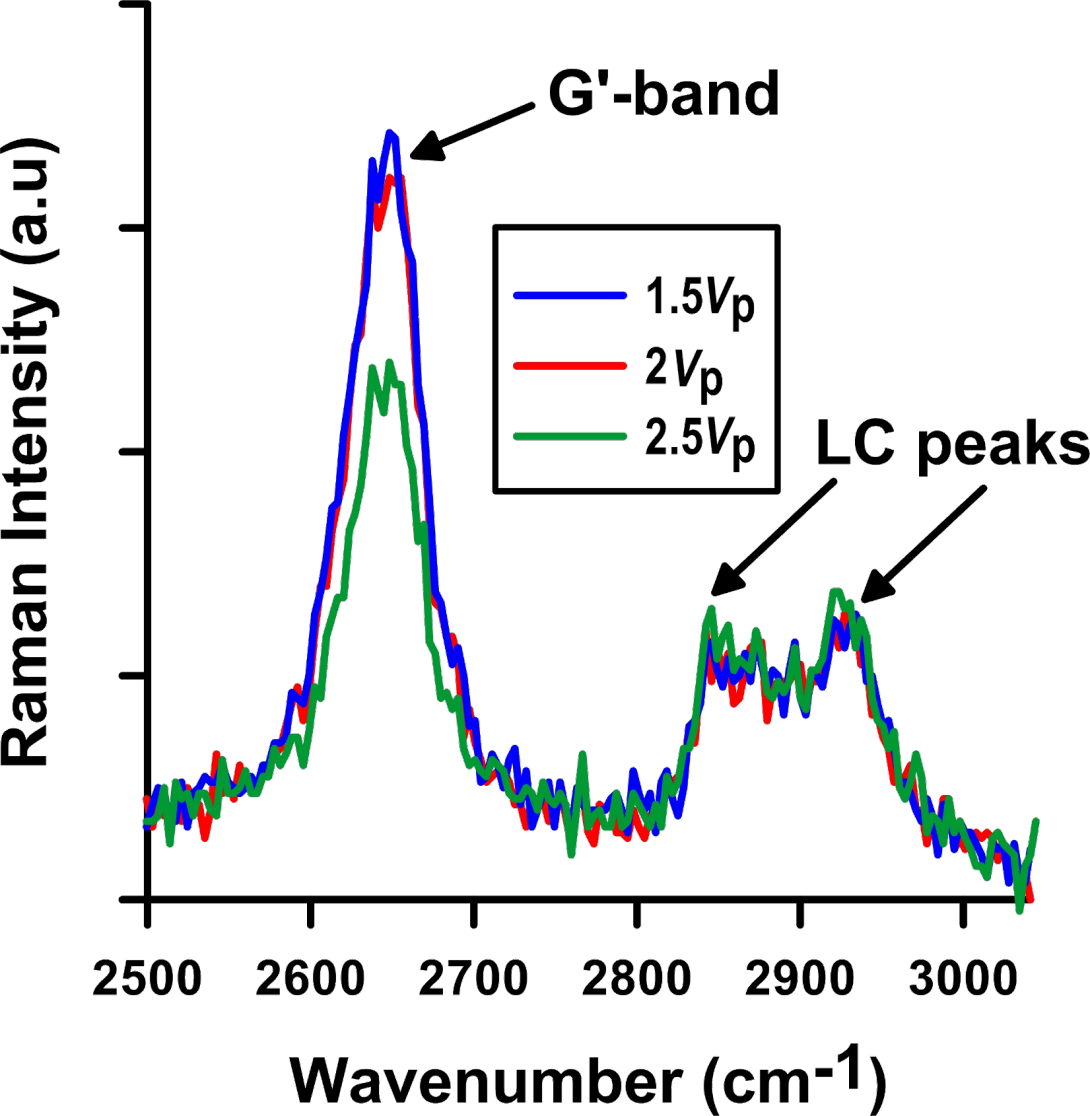
Raman Intensity evolution of the G’-band and LC peaks with voltage. The SWCNT threshold voltage is visible between 2*V*_p_ and 2.5*V*_p_.

Raman studies were performed on single, isolated SWCNTs in order to determine the individual threshold voltage (Figure 9). The Raman spectrum intensity remains constant at values under 2*V*_p_, but the SWCNT peaks begin to decrease at 2.5*V*_p_. Therefore, the threshold voltage in this case is between 2*V*_p_ and 2.5*V*_p_.

The discrepancy between the impedance measurements and the Raman study is because the latter considers small, isolated areas, while the impedance measurements are performed over the whole device. SWCNT aggregates increase the conductivity of the media [27] (i.e., decrease the impedance), but have a smaller relative surface, which would be less anchored by the LC and thus may “switch” earlier. Furthermore, the applied electric field also leads to larger aggregates [28] (in accordance with the percolation threshold theory). As aggregates accumulate, the sample conductivity increases even at a voltage lower than that at which the single SWCNTs reorient.

The threshold voltage can be directly linked to the interactions (i.e., anchoring) between the LC and the SWCNTs, much in the same way as the conventional Freedericksz transition voltage measured in conventional nematic LC cells. Furthermore, the anchoring causes the dispersed SWCNTs to relax in absence of an applied field. Since no electric field is aligned parallel to the glass slide, only the forces exerted by the aligned LC molecules cause the SWCNT to relax and return to a homogenous alignment.

## Conclusion

SWCNT switching in LC cells has been studied without observation of LC reorientation using Raman spectroscopy and impedance measurements. Using a negative anisotropic liquid crystal, in planar alignment, the LC molecules do not reorient, while the SWCNTs reorient from parallel to the substrate to perpendicular, depending on the applied electric field. This reorientation has been confirmed by monitoring the scattering in the vicinity, where the G-band intensity in Raman spectra are decreased and the impedance magnitude is decreased. This fact confirms that the SWCNTs reorient when they are immersed in LC due to the applied electric field and nonexclusively by the LC action. Well-dispersed SWCNTs showed reversible reorientation behavior while others (agglomerates) remained vertical after several excitation cycles. The agglomerates that were not reversible account for the overall system irreversibility detected by the impedance measurements. The observed threshold voltage, and the tendency of well-dispersed SWCNT relaxation, confirms the existence of the LC–SWCNT anchoring energy.

Pure LC materials are characterized by a very low conductivity. The doping of a LC material with CNTs leads to an increased conductivity, and thus an increased power consumption of any final device. Nonetheless, the doping of LC materials with CNTs provide important information on the LC–CNT interactions. CNT doping may be employed in vertically aligned cells with negative LC material in order to speed up relaxation, since the CNT will switch oppositely to the LC and provide a pseudo volume stabilization of the vertical alignment negative (VAN) structure.

## Experimental

The samples used for the impedance measurements were formed by two 0.7 mm thick ITO-coated glass plates (Glasstone) with resistivity of 100 Ω/□ and separated by 8.25 µm. The active area was 1 cm^2^. A LC planar alignment is induced by poly(3,4-ethylenedioxythiophene) and poly(styrenesulfonate) (PEDOT:PSS, Sigma-Aldrich). The PEDOT solution (1.3 wt % in water) was spin-coated at 1600 rpm and buffed.

Polyimides and polymers typically form the alignment layer to promote the planar orientation in the LC devices. Their use is not suitable for the impedance measurements because they provide insulation layers. PEDOT:PSS induces the planar alignment layer and is a conducting polymer, which is essential to keep electric continuity across the layers that form the LC cell.

The LC used for this study is the negative dielectric nematic mixture MLC-6608-000 (Merck). This LC has an optical birefringence of ∆*n* = 0.083 at 589.3 nm and is commonly used in VAN devices.

The LC is doped with SWCNTs (Unidym) with an outer diameter of 1 nm and a tube length of about 1 µm. The concentration of the SWCNT–LC mixture was approximately 0.001 wt %. A quantity of SWCNTs was previously diluted in toluene (0.1 wt %) and sonicated. After that, a portion of this solution was introduced in the LC. The SWCNT–LC mixture was sonicated and afterwards magnetically stirred at room temperature until toluene evaporation was complete. The LC cells are capillary filled.

The impedance measurements require a small signal (<0.1*V*_rms_) to obtain a linear system response. In this study, the effect of external electric fields must be included. Usually, the alternative current (AC) signal is set on a bias (offset) direct current (DC) voltage. But the use of DC voltages as a bias in LC materials is not recommendable because ion generation and migration lead to electrolytic degeneration. A low frequency (1 Hz) AC square wave is used instead. The AC wave amplitude varies according to the driving voltage under study. The LC reorientation is minimally affected by polarity changes by the use of a square signal. The low amplitude AC impedance probe signal (0.1*V*_rms_) is added. The details about the results sampling have been described in [15].

The Raman measurements are made using a visible wavelength Raman spectrometer (532 nm, spot diameter of 2 μm). A linearly polarized light beam was used for all measurements. The light impinges perpendicular to the LC cell surface and the linear polarization is parallel to the LC planar director. The amount of incident power is important because high power may cause optical trapping [29]. The optical trapping power depends on several factors (e.g., SWCNT length-to-diameter ratio, dispersed material) [30]. Therefore, the incident beam power was checked several times to avoid the optical trapping effect in this specific study. The average incident power used was 0.747 mW.
